# MCO Perspectives on Medicaid Policy: Racial Equity in Pregnancy and Child Health

**DOI:** 10.1089/heq.2024.0025

**Published:** 2024-08-07

**Authors:** Christine McClure, Cynthia Salter, Dara D. Méndez, Evan S. Cole, Sarah A. Sanders, Sydney Sharp, Marquita Smalls, Linda Adodoadji, Adena Bowden, Marian Jarlenski

**Affiliations:** ^1^ Department of Health Policy and Management, School of Public Health, University of Pittsburgh, Pittsburgh, Pennsylvania, USA.; ^2^ Department of Behavioral and Community Health Science, School of Public Health, University of Pittsburgh, Pittsburgh, Pennsylvania, USA.; ^3^ Department of Epidemiology, School of Public Health, University of Pittsburgh, Pittsburgh, Pennsylvania, USA.; ^4^ University of Pittsburgh Medical School, School of Medicine, University of Pittsburgh, Pittsburgh, Pennsylvania, USA.; ^5^ Healthy Start Incorporated, Research Department, Pittsburgh, Pennsylvania, USA.

**Keywords:** Medicaid, MCOs, maternal child health, health policy, health equity, qualitative methods

## Abstract

**Introduction:**

In 2020 and 2021, Pennsylvania implemented the Equity Incentive Program and the Maternity Care Bundled Payment program, two unique pay-for-performance (P4P) programs that provide financial incentives for managed care organizations (MCOs) that make improvements in utilization and quality metrics for Black women and children. The current study addresses gaps in the research about MCO perceptions regarding the ability of financial policy incentives to improve racial health inequities.

**Methods:**

Qualitative, semi-structured group interviews with representatives (*n* = 30) from the six Medicaid MCOs in Pennsylvania were completed in the summer of 2022. Data were thematically coded, using a preestablished codebook.

**Results:**

Interviews with representatives from six Pennsylvania MCOs generated four distinct but interconnected themes: (1) data optimism, (2) pursuing uniform care, (3) diffusion of responsibility, and (4) missing pieces of the puzzle.

**Discussion:**

Perspectives of MCO representatives indicate the need for MCO involvement in Medicaid policymaking. Interviews revealed MCO representatives’ perceptions that warrant further research: (1) the expectation for providers to change care delivery based solely on data, (2) racial health equity in pregnancy and child health can be accomplished by providing uniform care, and (3) the limited responsibility MCOs believe they have in addressing racial health inequities.

**Racial Health Implications:**

Little is known about MCOs’ general understanding of and reactions to P4P models and implementation, particularly models aimed at addressing racial inequities. Findings from this study can assist Medicaid agencies in understanding how MCOs interpret and implement equity-based policy to ensure intended populations are benefiting from the planned outcomes.

## Introduction

Despite being one of the wealthiest countries in the world, the United States continues to experience increasing maternal and infant mortality rates.^1,2^ In 2020, U.S. maternal and infant mortality rates were approximately three times those of most other high-income countries, at 24 maternal deaths per 100,000 births and 5.4 infant deaths per 1,000 births.^2^ Black women and infants represented the largest percentage of deaths, with Black women being over three times as likely to die from a pregnancy-related cause than White women and Black infants in the first year of life more than two times as likely to die than White infants.^3,4^ In 2018, Pennsylvania ranked 26th among U.S. states, with an overall maternal mortality rate of 82 deaths per 100,000 births; Black women were twice as likely as White women to die from a pregnancy-related cause, with a maternal mortality rate of 163 deaths per 100,000 live births.^5^ The infant mortality rate in Pennsylvania across racial groups was the highest for Black infants at 11 deaths per 1,000 births, compared with White infants at 4.5 per 1,000 births.^6,7^ According to the Pennsylvania Maternal Mortality Commission, decedents in more than half of the pregnancy-related deaths were enrolled in Medicaid.^8^

As the fourth-largest Medicaid program in the United States, with expenditures exceeding $35 billion and 3.5 million people enrolled, Medicaid policymakers have a unique opportunity to implement policies that address poor maternal health outcomes and promote racial health equity.^9–11^ Although policy interventions have been proposed to address racial inequities in maternal and child health, very few have been implemented.^12,13^

In a first step to achieve racial equity in pregnancy and child health, Pennsylvania Medicaid established two new policies. The Equity Incentive Program, implemented in 2020, makes available ∼$26 million in incentive payments for managed care organizations (MCOs) based on their annual improvement in quality metrics for prenatal care and well-child visits among Black families. Unlike traditional pay-for-performance programs (P4P) under which managed care plans’ performance is assessed overall, this program makes bonus payments contingent on improving timely access among Black beneficiaries. Separately, the Maternity Care Bundled Payment, implemented in 2021, is focused on reducing racial disparities for Black Medicaid beneficiaries across a range of pregnancy-related health outcomes. These models are similar to those in other states; however, these programs’ conditions shared savings for health care providers and MCOs who improve quality metrics specifically for Black beneficiaries. Other states, including Arkansas, California, Colorado, Connecticut, North Carolina, Tennessee, and Texas, have also implemented policies to improve racial health outcomes in their Medicaid programs; however, not all policies target a specific racial or ethnic group.^11^ Although the literature captures trends in P4P incentives, little is known about MCOs’ perceptions regarding these types of incentives and their ability to positively impact racial health disparities. The current study addresses gaps in the research about MCO perceptions regarding these two incentive policies and their ability to positively improve racial health inequities.

**FIG. 1.**
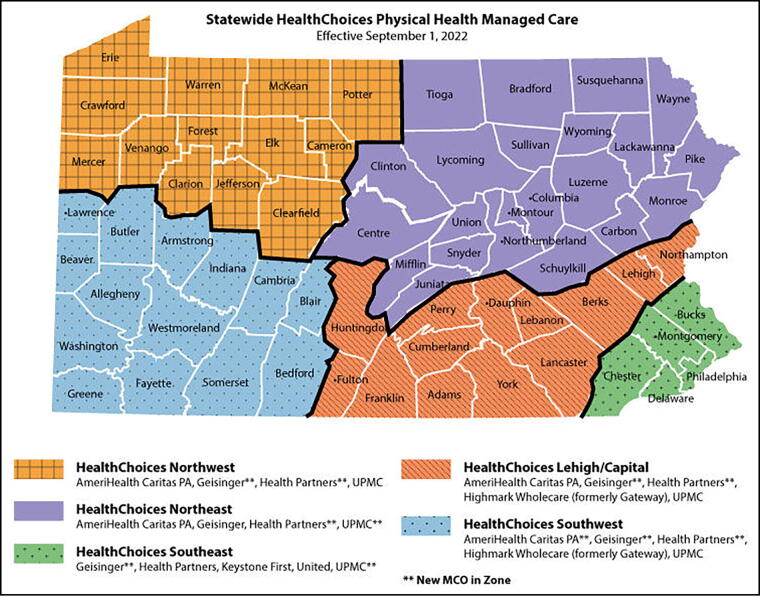
Managed care organization (MCO) coverage in Pennsylvania.

In Pennsylvania, HealthChoices is the program under which six MCOs are responsible for implementing the new P4P programs and administering Medicaid benefits (Fig. 1). To better understand implementation of the new incentive policies, our team conducted semi-structured interviews with representatives of the six Pennsylvania Medicaid physical health MCOs. We aimed to explore their perspectives of these Medicaid policies and their potential impact on racial health equity. This article includes the analysis of these qualitative interviews.

## Methods

### Study Design

This retrospective cross-sectional study collected qualitative data through six interviews with multiple representatives from each individual MCO. Theoretical constructs from Critical Race Theory, Public Health Critical Race Praxis, and literature at the intersection of race, class, beneficiary lived experience, and social welfare policy informed the interview guide.^14–20^ The interview guide was centered on three domains of racial health equity: (1) impressions of the equity-focused perinatal care bundle, (2) practice implications, and (3) policy implication. The interview guide was developed collaboratively by the research team, representatives from the state Medicaid agency, Department of Human Services, and community partners, including Healthy Start, Inc., Foundation for Delaware County, Hamilton Health, and the Maternity Care Coalition. The protocol included broad questions about the participants’ role in policy implementation and opinions on the policies’ impact on racial health equity, health practice, and future policy. The interview protocol is available in Supplementary Data S1. This study was reviewed and approved by the University of Pittsburgh, Institutional Review Board.

### Sampling and Recruitment

A purposive sampling strategy was used to recruit participants from each of the six MCOs in Pennsylvania.^21,22^ Partners from the Pennsylvania Department of Human Services assisted in connecting the study team with potential participants. Once identified, participants were recruited through email and asked to identify and invite other potential participants from their organization to participate in the interviews. All Medicaid MCOs agreed to participate, and each had at least three representatives participate in the group interviews. Interviews were scheduled via email, and questions and policy overviews were shared in advance.

### Data Collection

One-hour, semi-structured, remote group interviews were conducted using Zoom, in the summer of 2022.The study team elected to use a group interview format over focus groups, because the interview protocol was designed to have the participants respond to specific questions about decision making and the implementation of policy and not to elicit a group discussion the way that focus groups are designed.^23,24^ Before the interview, participants provided verbal consent, and no fewer than two research team members facilitated each interview. One research team member led the interview, whereas other team member(s) took field notes and asked clarifying questions when appropriate. After each interview, participants were asked to complete a demographic survey.

### Data Analysis

Audio-recorded interviews were transcribed verbatim by an external transcription service and stored in a secure, dual-authenticated, file-hosting service accessible only to the research team. NVivo 12, a qualitative analysis software, was used to manage and code interview transcripts.

To guide the coding process, the study team developed a codebook with specific codes related to the primary research questions and interview guide.^25^ Before the formal analysis began, two team members tested the codebook by independently coding one transcript after which they met to review their coding. A meeting was then held with the entire study team to discuss coding and to update the codebook. Study team members were then each randomly assigned two interview transcripts to begin the formal coding process.

Interview transcripts were thematically analyzed to identify patterns and themes across the data.^21–25^ During coding, each researcher took notes and created analytic memos to capture their thoughts and salient information. Throughout the process the study team met to discuss themes and to update the codebook when necessary. After coding was completed by all study team members, one team member conducted a separate thematic analysis of all six interview transcripts to further triangulate the data and to ensure that saturation was reached.^23–25^ This separate thematic analysis was also guided by the study’s codebook but was hand-coded in Microsoft Word rather than in NVivo.

## Results

Thirty participants across all six Pennsylvania Medicaid physical health MCOs participated in group interviews. See Table 1 for demographic and professional information about the participants. Most of the participants identified as White (*n* = 18), female (*n* = 19), director/manager-level employees (*n* = 13), with more than 20 years of experience in health care (*n* = 17).

**Table 1.** Demographic and Professional Information of MCO Group Interview Participants (*n* = 30)

Variable	*n* (%)	Missing *n* (%)
Gender		7 (23.3%)
Female	19 (63.3%)	
Male	4 (13.3%)	
Race		7 (23.3%)
African American	2 (6.7%)	
Asian	3 (10.0%)	
White	18 (60.0%)	
Ethnicity		7 (23.3%)
Hispanic	1 (3.3%)	
Indian	1 (3.3%)	
MCO		0 (0.0%)
AmeriHealth Caritas	4 (13.3%)	
Gateway/Highmark Wholecare	4 (13.3%)	
Geisinger	3 (10.0%)	
Health Partners	5 (16.7%)	
United Health care	3 (10.0%)	
University of Pittsburgh Medical Center (UPMC)	11 (36.7%)	
Title		3 (10.0%)
High-level executive (e.g., VP)	5 (16.7%)	
Senior director/Manager	6 (20.0%)	
Director/Manager	13 (43.3%)	
Other*	3 (10.0%)	
Years in health care		7 (23.3%)
<10	2 (6.7%)	
10–19	4 (13.3%)	
20–30	9 (30.0%)	
>30	8 (26.7%)	

*Other position titles.

MCO, managed care organization; UPMC, University of Pittsburgh Medical Center.

At least three representatives from each Medicaid MCO (*n* = 6) participated in group interviews. The average group size was five participants (*n* = 5) with interviews ranging from 33 to 59 min and averaging 47 min.

### Themes

Four distinct but interconnected themes were identified in the data: (1) data optimism, (2) pursuing uniform care, (3) diffusion of responsibility, and (4) missing pieces of the puzzle.

#### Theme one: Data optimism

Data was one of the most highly discussed topics during the interviews. Participants from all MCOs described the importance of using all available data (i.e., MCO data, Medicaid claims data, electronic health records data) to identify and develop strategies to align with the new policies and focus on addressing racial and ethnic health disparities for Black women and children in Pennsylvania.

“[I]f we don’t look at the data, we can’t find the disparity, and we can’t solve for it … data is key … data is a very important part of the conversation …” (MCO #4)

Using data to raise provider awareness about racial health inequities, including presenting baseline data to providers, disaggregated by race and ethnicity, was also discussed.

“… we work with the provider … we engage them … we give them meaningful reports … we show them the data. historical data … create a baseline of where they started … we show them tools and areas of opportunity … for transformation.” (MCO #1)

Participants from multiple MCOs described expectations for providers to change practice after seeing the racial inequities in the data.

“… when we presented the data to providers, it showed significant disparities … we’re hoping that that kind of changes the way that providers are providing care…” (MCO #2)

One participant said they previously used an approach of showing data to spur action, but this was not successful so they began focusing on the incentive:

“We’ve been offering detailed and summary statistics … around health equity … the experiences for their members of color … that alone wasn’t enough to encourage action. So, data alone isn’t leading to movement from providers … having an incentive … we’re optimistic about the impact …” (MCO #4)

Improving data quality was noted as one of the priorities related to implementing the new policies, with eight participants describing data integrity, lack of confidence in the data, and providers not capturing race and ethnicity as their most significant data challenges.

“… one issue was the data … we’re still in the process of improving the way that we collect this data. It’s … not clean … that’s critical … to accurately collect that data.” (MCO #2)

#### Theme two: Pursuing uniform care

When asked about the purpose and the policies’ ability to impact racial equity, participants often described programs that focused on all patients and not a “specific community.” “Pursing uniform care” is the idea of providing uniform approaches and practices regardless of the historical and existing health inequities. The participants discussed the creation of standardized processes and protocols that would focus on “everyone across the board” and the importance of “reaching the goal of uniform care” in the following quotes:

“… we focus on everybody as a whole, not necessarily demographics per se.” (MCO #3)

“… when you put in all these standardized processes and protocols and pathways the same … you tend to see the same … greater level of care … across the board … that does help improve equity.” (MCO #4)

Only one participant from MCO #4 described the connection between health equity, social determinants of health (SDOH) and structural racism:

“… for anything health equity … health equity is intimately related to the social determinants of health, and it’s intimately related to structural racism.” (MCO #4)

The need to consider SDOH and to move away from uniform care as the sole approach was also suggested:

“… these models allow us to say why it’s important to look and that it really isn’t a one size fits all … you do have to incorporate … social influencers that really affect their care.” (MCO #4)

#### Theme three: Diffusion of responsibility

Although participants were not asked specifically about accountability or responsibility, in promoting health equity, participants described a shared responsibility to improve health inequities, although attributing most of the responsibility to members (patients), providers, and community-based organizations (CBOs), and not MCOs:

“… you want to make everybody … accountable for the care, including the member … The member has to be accountable … provider has to be accountable … we have to be accountable to be able to help and orchestrate all that. So, it’s a true collaboration.” (MCO #1)

“… with the black population is culturally, they’re more raised to … only seek healthcare when there’s a problem or an issue; it’s not so much seen as a preventative.” (MCO #2)

“… and my hope is that the providers will have better insight … as we break out that data… So, I’m hopeful that it will be a little more eye-opening and they’ll pay more attention.” (MCO #6)

The below quotes from participants describe the role of CBOs in achieving policy goals and being gatekeepers in minoritized communities, especially to address structural and SDOH.

“… every market that we’re in, there’s community groups … They really serve as sort of that voice, that understanding of how services are experienced and how they can be improved.” (MCO #2)

“… achieving health equity is couched in addressing community needs … addressing day-to-day life needs … that’s further connection with CBOs … connecting providers to the CBOs in their communities.” (MCO #4)

“… also listening to the communities … the data will tell us one piece … it’s also understanding what the community needs are … what the community-based organizations are hearing and seeing…” (MCO #5)

#### Theme four: Missing pieces of the puzzle

When asked how these policies would affect health inequities or how they could be improved, participants recommended extending Medicaid coverage for at least one year postpartum, increasing clinician engagement beyond the practice leadership level, considering nonutilization measures for determining service needs, integrating behavioral health services, and modernizing the Medicaid application process to capture and share the cell phone and email data of members during enrollment. The below quotes reflect these recommendations:

Expanding Medicaid coverage:

“… expanding Medicaid for a full year into the postpartum period … so much time taking care of these mothers during the pregnancy and then 60 days after they deliver—they’re off the radar.” (MCO #1)

Improving access and improving quality of care:

“… the way this policy is written … it’s not measuring the actual quality of care … experienced by the person in the office, which is where the breakdown occurs … if you’re only measuring can I get to that care … you’re missing 90% of the puzzle.” (MCO #5)

Integration of behavioral health services with maternity and postpartum and modernize the Medicaid application process:

“… the behavioral health component … that’s such a big portion … incorporating that throughout the entire pregnancy as well as in the extended period …” (MCO #1)

“… we are in 2022, how can the state help us acquire cell phone data and email data for our members as part of the application process … parents want to engage with us using cell phones and smartphones … having access to that information is critical importance for these interventions.” (MCO #4)

Table 2 provides additional exemplar quotes to support the above themes.

**Table 2.** Additional Exemplar Quotes

Theme	Exemplar quotes
Data optimism	“… I have noted in speaking to the providers and the practices is that some of them may not be collecting the race and ethnicity and language in their EMR system.”
	“They don’t have the ability to even pull that type of data to pull here are all my African American pregnant moms … so again that is something that a lot of EMR systems are not able to do and they are working on that, the providers but they actually accept our information at this time.”
	“… we are able to share their won patient data … some of them don’t even know … if they don’t know it makes it difficult to implement programs … to make some decisions about how they can improve some of those metrics, especially around equity.”
	“… the reason you give them the baseline data is to show them areas of opportunity and then you want to see how that changes on a quarter by quarter basis.”
Pursing uniform care	“By providing the providers with adequate data and our input as well as hopefully reaching the goal of uniform care throughout the platform regardless of race and age. We feel we’re going to elevate some of the inequities to a level playing field …”
	“… the goal of the bundled payment is really just to encourage providers to increase the quality of care that they’re providing to members and patients …”
	“I think with the ability to collect that data … there will potentially be an increase in trying to ensure that our members just across the board are coming in for their visits …”
	“… I think it will help our entire maternity population, but also the African American because it’s a big chunk of our population …”
Diffusion of responsibility	“… we’re really missing out on the members who are not engaged at all in their care …”
	“… I am often asked, and its usually with a tone of indignation. What a woman could possibly find more important that going for her prenatal visit, and my answer is something else …”
	“… our patients perspectives need to factor into all of this because I think we as clinicians come up with a lot of great ideas, but if we are not checking in with what the community is saying … we may be completely missing the mark.”
	“The provider has to be accountable to let us know and look at the information and see what they can do … we have to help orchestrate all that … so it’s a true collaboration.”
Missing pieces of the puzzle	“Most of the clinicians are not at the table, are not event aware this is a value-based program … what I like about the maternity care bundle, that the money has to go towards the local providers … because I always feel like I’m the only clinical at the meetings … there’s no clinicians at the table.”
	“… I think these policies are created within a system that’s very much looking at one specific type of measurement … If you are only measuring access, that’s not the point of it all.”
	“The physicians … midwives, providers, nurse practitioners are the ones who are actually in the rooms, the ones who are actually providing all that care, they are not receiving the feedback on how they are doing … I just see the patients and then the system reaps the benefits …”
	“I would say we need to think globally and act locally in resolving disparities.”

## Discussion

This study provided insight into Medicaid MCO’s perceptions of two Medicaid policies, the Equity Incentive Program and the Maternity Care Bundled Payment program. Participants shared their experience regarding the variation in data collection capacity and usage, the role of data in advancing racial equity, challenges in using data to raise provider awareness, and optimism that data showing inequities would motivate providers to change their care delivery practices.

Although the P4P literature highlights the importance of data to inform improvements in health care quality, strategies that focus on data-driven outcomes like the ones discussed by the participants rather than those that are clinically significant have been shown to be less successful.^26,27^ Discussion during the group interviews focused more heavily on improving data quality to measure patient access rather than how data could be used to achieve the policies primary goal of improving health outcomes for Black women and children.

The P4P literature also underscores other topics such as the size of the incentive, alignment with organizational goals, and resources needed to implement the policies. These topics were minimally discussed during the group interviews, as participants were not asked questions in these areas; therefore, it is unclear how the MCOs are handling these issues.

Research indicates that social and systemic barriers, including systematic racism, exposure to racial trauma, marginalization, and implicit bias within the health care system, are major contributors to poor outcomes for Black pregnant women and their children, including decreased engagement with perinatal care.^28^ Despite this, only one participant referenced the need to confront structural racism to improve outcomes in this population. Moreover, when participants were asked questions about racial health equity, the discussion shifted toward SDOH, the role of CBOs, and the need for quality data to decrease existing health inequities. Participants did not describe strategies to address the social and systemic issues that are the foundation of racial inequities.

Although the participants understood the role of SDOH in exacerbating health inequities, some of the interventions described during the group interviews were not explicitly focused on Black pregnant women and children. This is relevant because the new policies specifically focus on improving maternal and child health outcomes for Black populations. When discussing specific efforts or programs, participants from the six MCOs shared examples of existing or forthcoming case management strategies targeting all patients, with only two describing programs specifically for Black women and children. These uniform approaches toward improving outcomes commonly discussed in the group interviews by some MCOs do not align with developing solutions tailored to the unique needs of specific populations, including Black pregnant women and children.

### Strengths and Limitations

This study was conducted early during the policies implementation which could result in the participants’ limited experience with the policies. As part of a larger mixed-methods study, these group interviews capture only the perspectives of MCO representatives and not the perspectives of clinicians and Medicaid recipients. However, future work will involve additional qualitative studies with Medicaid recipients, clinicians, and birth workers. Study strengths include a robust qualitative interview guide and analytic process that was informed in collaboration with community and state Medicaid partners across the state. The study included representation from all MCOs across Pennsylvania. Although the pandemic has influenced policy roll-out and implementation, this study captures MCOs understanding the policies and their early phase approaches to implementation.

## Health Equity Implications

Although participants clearly understood that the new incentive policies were connected to improving health equity for Black pregnant women and children, the group interview discussion centered largely on improving data quality, the implementation of programs that would benefit all pregnant women, while undervaluing the social and structural barriers that reinforce health inequities observed across the health care system. Findings from this study contribute valuable feedback to assist Medicaid agencies in understanding how MCOs interpret and implement equity-based policy as they continue to improve existing and develop new policies to reduce health inequities.

## Abbreviations Used

MCO: Managed Care Organization
P4P: Pay for Performance
U.S.: United States
