# Supplementary material for: MCO Perspectives on Medicaid Policy: Racial Equity in Pregnancy and Child Health
Source: Health Equity. 2024 Aug 7;8(1):505–12. doi: 10.1089/heq.2024.0025 (PMC11347871; doi:10.1089/heq.2024.0025)
Supplement: Supplementary Data S1 [file heq.2024.0025_mco_interview_guide_v2.pdf]

Implementing and evaluating structural interventions in Medicaid to promote racial equity in pregnancy and child health

MCO Verbal Interview Guide

INTRODUCTARY SCRIPT

Good afternoon, I am [*insert name*]. I am part of a research team at the University of Pittsburgh School of Public Health, and I am going to be leading our interview today.

As you may know, Pennsylvania Medicaid recently adopted a maternity bundled payment policy that has a component focused on improving pregnancy outcomes for Black people, as well as incorporating an equity component into the pay for performance program. The purpose of this interview is to learn about the perspectives of Medicaid managed care organizations regarding these new Medicaid policy, and to understand perspectives about how managed care organizations can promote racial equity in care. If you are willing to participate, we're going to talk for up to 1 hour. We will ask you several open-ended questions about your experiences, and we will also ask you your age, sex, race, and years working in the field of Medicaid managed care. There are no foreseeable risks associated with this interview, nor are there any direct benefits to you.

Remember that everything that is said in this interview is confidential. All responses are confidential and the results will be kept on a secure server in a password-protected computer and locked office. Your research data may be shared with investigators conducting similar research; however, this information will be shared in a de-identified manner (without identifiers). Your participation is voluntary, and you can withdraw from the study at any time. This study is being conducted by Marian Jarlenski and Dara Mendez, who can be reached at 412.383.5363 if you have any questions.

It is important that you understand that there are no right or wrong answers for any of the things we are going to talk about. Your ideas are extremely valuable, and I'm interested in <u>your</u> comments and opinions. The goal of our study is to better understand the impact of these policies on healthcare providers and patients in order to improve policies and protocols. Do you have any questions? Do you agree to participate in this interview? Do you agree to be audio recorded?

I am going to record the discussion. Everything you say is important to us, and we want to make sure that we do not miss any of your comments. Because we are recording all interview responses anonymously, we will give a random study ID number to this interview. This random ID number will help us identify each interview when we analyze the data at a later time. OK, now we will begin.

Domain 1: Impression of the equity-focused perinatal care bundle

First, let's review this policy. I will read you a brief description of the policies.

Under maternity care bundled payment models, obstetric providers are incentivized to meet a total cost threshold and quality metrics for prenatal and delivery care. Specifically, providers and payers agree on a target cost for a low- or average-risk maternity care, including pregnancy, delivery, and postpartum care. If total payments to providers are lower than the target cost while maintaining certain quality metrics, providers and payers share those savings. In addition to these shared savings, providers are eligible for additional incentive payments if the quality metrics for Black patients exceed the 75th percentile of the national performance.
Performance metrics include:

- Early prenatal care initiation
- Social Determinants of Health screening
- Prenatal depression screening and follow-up
- Prenatal immunizations
- Treatment for substance use disorders
- Postpartum care visit
- Postpartum depression screening and follow-up
- 2+ well-child visits during first 60 days of life

Currently, the Pennsylvania Medicaid program makes available 2% of total payments to MCO plans, contingent on MCO plan performance on 13 different healthcare quality metrics. Starting in 2020, an equity incentive payment program was added to this reimbursement scheme for 2 metrics: timely prenatal care and well-child visit utilization in the first 15 months of life. Specifically, 2/13 (or 0.15%) of total payments are withheld. MCO plans are assessed on overall performance and subsequently on the annual improvement on these measures among Black beneficiaries. MCO plans can receive a range of payment adjustments (ranging from -0.12% to +0.35% of their total payments).

1) Can you describe your role in implementing either or both policies? (

If interviewee is working on the maternity bundled payment model, ask the following:

2) Please take a moment to comment on this bundled payment model.
   I. PROBE: What do you believe is the policy goal of this model?
   II. PROBE: What factors might facilitate the model?
   III. PROBE: What factors might be barriers to this model?

3) In your opinion, how might this new payment model spur changes to promote racial equity?
   I. PROBE: Do you feel it will promote racial equity?
   II. PROBE: What are healthcare systems barriers/facilitators to racial equity?

If interviewee is working on the equity incentive payment program, ask the following:

4) Please take a moment to comment on equity incentive payment policy.
   IV. PROBE: What do you believe is the policy goal of this model?
   V. PROBE: What factors might facilitate the model?
   VI. PROBE: What factors might be barriers to this model?

5) In your opinion, how might this new payment policy spur changes to promote racial equity?
   III. PROBE: Do you feel it will promote racial equity?
   IV. PROBE: What are healthcare systems barriers/facilitators to racial equity?

Domain 2: Practice Implications

If interviewee is working on the maternity bundled payment model, ask the following:

1) What kind of changes do you perceive providers might make under the payment model?
    I. PROBE: Rate your view of the importance of this policy on health outcomes from a scale of 1 to 5 with 1 being the lowest and 5 being the highest.
    II. PROBE: What implications do you think this kind of payment reform has for perinatal care generally
2) What about equity in pregnancy and birth outcomes – What kind of changes do you perceive providers might make to advance racial equity?
    i. PROBE: What kind of data do you normally have on the race of your plan members?
    ii. PROBE: Comment on the benchmark: outcomes for Black patients at the 75th percentile of the national average.

If interviewee is working on the equity incentive payment program, ask the following:

3) What kind of changes, if any, is your plan undertaking in response to the equity incentive payment program?
    i. PROBE: Comment on the amount of the incentive – is it the right amount – compare/contrast to other incentive programs.
4) What are barriers or facilitators to improving the metrics for Black patients?
    i. PROBE: Comment on the amount of improvement required under the policy.
    ii. PROBE: Comment on the metrics (HEDIS metrics for timely prenatal care and well child visits).

Domain 3: Policy Implications

1) Please list the ways, if any, that you would recommend changing PA's policy.
   Note: Text of PA Policy is available to review upon request.
   PROBE: What is your hope for these types of policies in terms of what they might accomplish?

2) Please take describe your thoughts on the right level of policy change (if any) to advance health equity.
    I. PROBE: Hospital, County, MCO, Regional, State, Federal

DEMOGRAPHICS SURVEY

Random Study ID: ________________________________

Question	Answer
Age	
Gender	
Race	
Years working in healthcare	
Professional title	
